# deepNGS navigator: exploring antibody NGS datasets using deep contrastive learning

**DOI:** 10.1093/bioinformatics/btaf414

**Published:** 2025-08-11

**Authors:** Homa MohammadiPeyhani, Edith Lee, Richard Bonneau, Vladimir Gligorijevic, Jae Hyeon Lee

**Affiliations:** Prescient Design, Genentech; Prescient Design, Genentech; Prescient Design, Genentech; Prescient Design, Genentech; Prescient Design, Genentech

## Abstract

**Motivation:**

High-throughput sequencing uncovers how B-cells adapt in response to antigens by generating B-cell-receptor (BCR) sequences at an unprecedented scale. As BCR datasets grow to millions of sequences, using efficient computational methods becomes crucial. One important aspect of antibody sequence analysis is detecting clonal families or clusters of related sequences, whether they come from immunization, synthetic-libraries or even ML-generated datasets.

**Results:**

We introduce deepNGS Navigator, a computational tool that leverages language models and contrastive learning to transform antibody sequences into intuitive 2D representations. The resulting 2D maps offer a visualization of overall diversity of input datasets, which can be clustered based on the sequence distances and their densities across the map. Beyond grouping related sequences, the 2D maps also represent mutational patterns inferred from sequence embeddings, enabling trajectory analysis and clustering within the projected space. By overlaying properties such as charge, the map helps identify clusters of interest for further investigation while also flagging potentially noisy or non-specific sequences with higher risk. We demonstrate deepNGS Navigator’s utilities on several datasets, including: (i) a synthetic-library from a yeast-display targeting HER2, (ii) a machine learning-generated dataset with a hierarchical structure, (iii) NGS sequences from a llama immunized against COVID RBD, (iv) human naive and memory B-cell sequences, and (v) an *in silico* dataset simulating B-cell clonal lineages.

**Availability and implementation:**

The deepNGS Navigator source code is available at: github.com/prescient-design/deepngs-navigator and github.com/prescient-design/deepngs-navigator-panel-app.

## 1 Introduction

**Figure 1.**
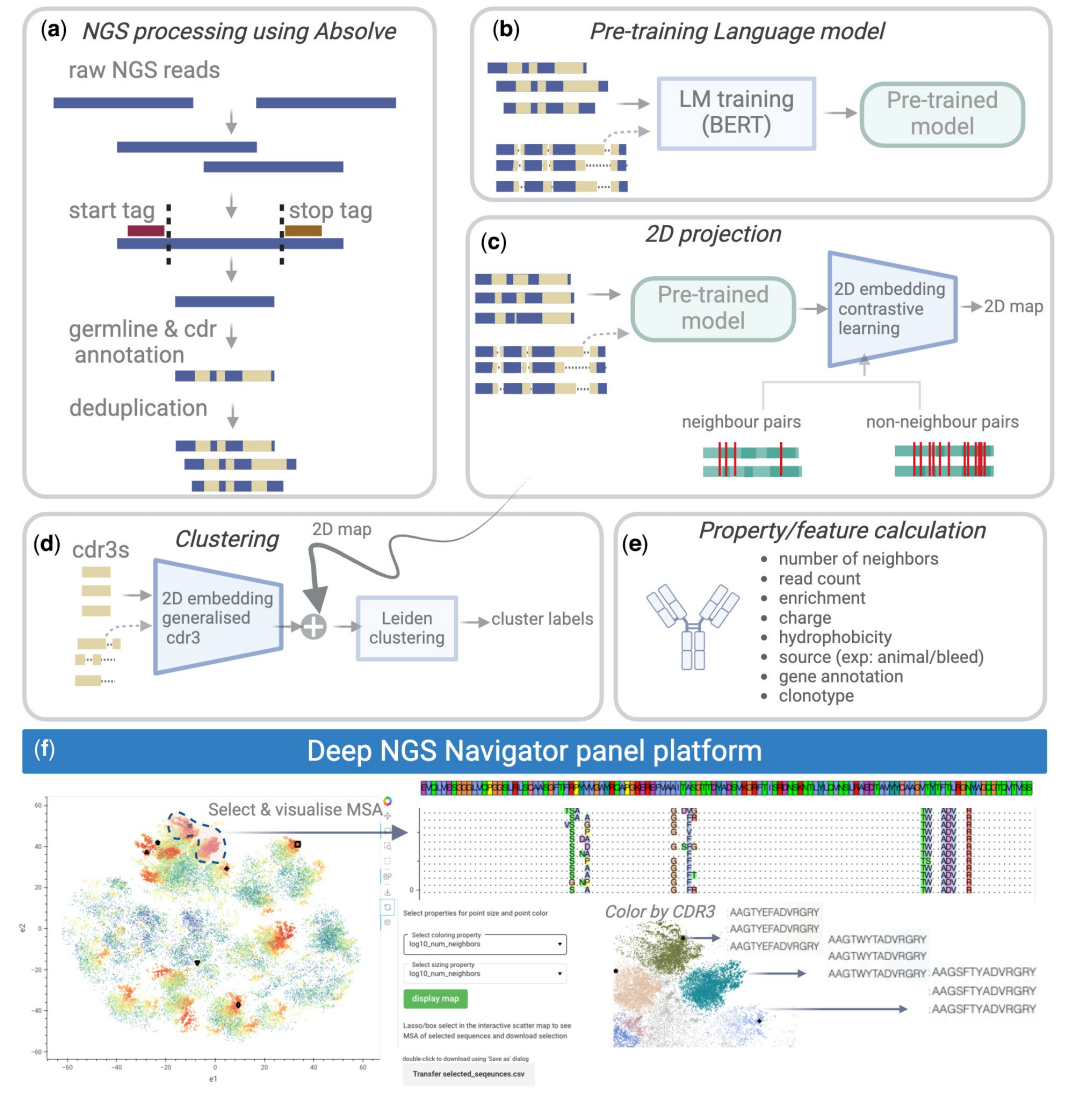
deepNGS Navigator workflow. (a) Preprocessing raw NGS datasets using Absolve, including merging forward and reverse reads and optionally translating them into proteins (or users can directly cluster nucleotide sequences). The sequences are annotated by their CDR3, deduplicated, and their copy number is recorded for each unique sequence. In the final table each row represents a unique sequence with minimum heavy and/or light chain and their respective CDR3 information. (b) Pretraining a language model on preprocessed NGS datasets by using either aligned sequences or groups of sequences with same sequence and CDR3 lengths. (c) 2D projecting using LM and contrastive learning, where neighbors and non-neighbors for each sequence are defined based on allowed edit distance in CDR3 and full sequence. (d) Assigning cluster labels using Leiden algorithm. Optionally edge cases of each cluster can be refined using CDR3 embedding of sequences. (e) Further annotating dataset by calculating various properties and features, including charge, hydrophobicity, and neighbor count. (f) Interactive visualization of final 2D map in deepNGS panel platform, allowing users to color the map based on various properties, select sequences of interest and plot their multiple sequence alignments (MSA). Created with BioRender.com.

Advances in high-throughput sequencing technology have significantly enhanced our ability to investigate large biological and synthetic antibody libraries, providing opportunities to harness deep insights into immune response and underlying patterns. However, analyzing and interpreting the large volume of complex data generated from immunization or synthetic B-cell libraries poses a significant challenge (Gallo 2024, Wossnig *et al.* 2024). Though phylogenetic tree based methods provide valuable insights into the clonal evolution of B-cells, their application to large-scale B-cell repertoires is limited by computational scalability, incomplete germline databases of camelids (Hanke *et al.* 2022), complex gene conversion mechanisms in rabbits (Lavinder *et al.* 2014), and a requirement on the minimum number of sequences within each clonal group for meaningful analysis, which often restricts analyses to only the largest V(D)J gene groups (Yermanos *et al.* 2017, 2018, Nouri and Kleinstein 2018, Lindenbaum *et al.* 2021). Machine learning (ML) methods for 2D visualization of sequences like UMAP (McInnes *et al.* 2020) are sometimes used to enable visual exploration of sequence dataset structure (Hanke *et al.* 2022). However, it is often unclear how to interpret and use such projection maps for practical applications, such as clonotyping and hit selection or for follow-up experimental characterizations. To address these challenges, we developed deepNGS Navigator that leverages deep learning based language models (LM) and contrastive learning to transform high-dimensional antibody sequence repertoire data into intuitive 2D maps (Fig. 1). These maps enable clustering of sequences with shared mutational profiles and approximate underlying evolutionary relationships directly from raw sequence data. By providing an intuitive, scalable view of repertoire structure without relying on germline references, deepNGS Navigator facilitates downstream analyses and informed selection of diverse, functionally distinct candidate sequences for follow-up studies.

deepNGS Navigator takes NGS datasets (nucleotides or amino acids) as input (Fig. 1a). It utilizes a BERT-type language model (Devlin *et al.* 2018) to embed sequences into high dimensional feature space, which can represent complex relationships and patterns among input sequences (Fig. 1b). Then using a contrastive learning technique, inspired by frameworks like SimCLR (Chen *et al.* 2020) and t-SimCNE (Böhm *et al.* 2022), the high dimensional LM embedding is projected to 2D maps (Fig. 1c). As part of contrastive learning process, neighbors and non-neighbors for each sequence are defined based on allowed edit distance in both the complementary-determining regions 3 (CDR3) and the full sequence, tailored specifically for antibody sequences. Next, Leiden algorithm (Traag *et al.* 2019) is used on the 2D projection to identify clusters. Given the importance and diversity of the CDR3s, users have an option to incorporate additional embeddings of CDR3s at a lower weight to further refine the maps (Fig. 1d). Finally, users can annotate the dataset with various metrics, such as biophysical properties like charge and hydrophobicity, and then interactively explore the 2D maps within the deepNGS Panel platform. This includes drawing, selecting, visualizing MSAs(as a follow-up tool for detailed inspection), and downloading selected sequences (Fig. 1e and f). To showcase the method’s broad applicability, we used deepNGS Navigator to analyze following datasets from various studies:

1. We generated 2D maps of synthetic library sequences from a yeast display annotated by FACS binding labels to HER2. The results of the analysis show that our approach produces clusters with smaller average pairwise edit distances and lower label entropy compared to alternative methods explored.
2. We analyzed synthetic machine learning-generated sequences with a hierarchical tree structure rooted on a “seed” sequence. Our analysis shows that the deepNGS Navigator method provides an embedding map whose global organization captures the edit distance relationship better than that of alternatives.
3. We clustered NGS sequences from a llama immunized against COVID RBD and show that the method achieves fewer, larger clusters with comparable enrichment purity compared to a prior method.
4. We applied the visualization to a dataset consisting of naive and memory derived human B-cells and show the proximity of sequences in the embedding map is correlated with their potential biological relations.
5. We reconstructed clonal families in an *in silico* designed dataset and demonstrated that deepNGS performs similar to the state-of-the-art methods in accurately identifying lineages. Moreover, it shows superior performance in separating true lineages from noisy datapoints, while relying solely on sequence information without gene annotation guidance.

These applications highlight the potential of deepNGS Navigator to transform complex sequence spaces into intuitive 2D maps, that facilitate discovery workflows by clustering sequences with similar biological functions.

## 2 Materials and methods

### 2.1 Data collection and initial processing

Pre-processing antibody NGS datasets includes the following steps: (i) merging raw forward and reverse reads, (ii) translating sequences to protein, and (iii) performing germline annotation. In this study, raw datasets were pre-processed using Absolve https://github.com/Genentech/Absolve. Output tables from Absolve were further refined by correcting sequence prefixes and suffixes to reduce primer contamination based on their germline information. Then, sequences were deduplicated, while keeping read count as an attribute for number of occurrences of each sequence in dataset. Germline annotation [step (iii)] can be useful for sanity checks of the final maps, but it is not mandatory. Translating sequences to proteins in step (ii) is also optional. As demonstrated in our last case study, deepNGS can effectively analyze nucleotide sequences as well.

deepNGS Navigator expects input data structured in a tabular format with each row representing a unique sequence, containing the following columns:

- fv_heavy: heavy chain sequence.
- fv_light: light chain sequence.
- HCDR3: heavy chain CDR3.
- LCDR3: light chain CDR3.

If one chain is missing, its respective column can be left empty. Based on the case study and available data, users can choose to apply deepNGS to all input sequences with varying lengths, resulting in a single comprehensive map for all of them. In this scenario, it is necessary to align the sequences using methods like AbAlign (Zong *et al.* 2023) or AHo numbering (Honegger and Plückthun 2001). Alternatively, for more granular embedding maps, users can group sequences by their sequence and CDR3 lengths, and optionally by V-gene, and generate 2D maps specific to each group.

### 2.2 Language model training and embedding sequences into 2D space

Language models (LM) have been shown to capture complex patterns in biological sequence data (Li *et al.* 2021, Santuari *et al.* 2024, Zheng *et al.* 2024). In deepNGS Navigator, by default, a BERT type LM (Devlin *et al.* 2018) is pre-trained on all input antibody repertoire data. When working with a small dataset, one can replace this step by using LMs pre-trained on larger, more general datasets such as AbLang (Olsen *et al.* 2022), which is specifically trained on antibodies. Next, we use contrastive learning on high dimensional embeddings of sequences to project them to 2D maps. Specifically, we draw inspiration from SimCLR (Chen *et al.* 2020), and adapt the recently introduced t-SimCNE method (Böhm *et al.* 2022), which has shown promising applications in analyzing and clustering image data.

The architecture of deepNGS Navigator model consists of three main parts:

- A LM component with BERT-style architecture, initialized from a pre-trained model, which generates high dimensional embeddings of each sequence (with a default size of 256). As a result, regardless of the original sequence length, the component maps all sequences to a fixed-size vector.
- A linear transformation layer that accepts the LM embeddings as input and outputs embeddings of the same dimension.
- A final linear projection head that projects the high dimensional embeddings to a 2D space.

### 2.3 Neighbor definition

Our contrastive learning methods require the definition of positive and negative sequence pairs. Here, neighbors and non-neighbors. In this work, we define neighbors based on Hamming distance. Sequences within a defined Hamming distance cutoff are considered neighbors. Users can either set aHamming distance cutoff or suggest an estimated number of neighbors that each sequence should have on average. This estimate is then used to calculate the appropriate Hamming distance cutoff. This is especially useful when analyzing datasets with high diversity where it is challenging to define reasonable cutoffs in advance. For an efficient clustering using contrastive learning regardless of the dataset, we recommend each sequence to have an average of at least 5–10 neighbors. Achieving this number of neighbors may require a different Hamming distance cutoff depending on the dataset. We suggest starting with this range and adjusting the cutoff as needed, either tightening or relaxing it based on the results. In antibodies, CDR3 similarity is particularly important for maintaining functional relevance. To take this into account and control the CDR3 diversity among neighbors, we set a separate threshold on Hamming distance specifically for the CDR3 region. This threshold can be defined manually or adapted on a dataset basis as mentioned above. The default Hamming distance cutoff is 5 for the full sequence, with a maximum 2 out of 5 in the CDR3 region, unless specified by user input. As an important note, sequences that do not meet the minimum requirement of having at least 5 neighbors are excluded from training by default. This exclusion criterion is helpful as sequences outside of it have difficult-to-identify placements on the map and often tend to obscure relations of other sequences with more neighbors by acting as background noise. Using biologically meaningful neighbor definitions and adaptive cutoff strategies ensures stable, reproducible (despite possible rotation or flipping) and interpretable maps that minimizes visualization bias.

### 2.4 Optimizing loss function for 2D embeddings

We utilized the Cauchy similarity loss inspired by t-SimCNE with a few modifications that allow users to experiment and adjust depending on the case study. Given a batch of *N* sequences ${\left\{x_{i}\right\}}_{i=1}^{N}$, we embed each sequence into a *d*-dimensional latent embedding $e_{i}\in R^{d}$ using a LM $f:x_{i}\;\mapsto \;e_{i}$. For each sequence $x_{i}$, we randomly select and embed a single neighbor $x_{i}^{\prime }$ from its precomputed list of neighbors, resulting in 2 *N* embedded sequences.

Next, we compute the following pairwise Euclidean distances between embeddings:

1.
$d_{ij}^{\left(11\right)}$ : the squared Euclidean distances between all pairs of original sequences, defined as:
  $$d_{ij}^{\left(11\right)}=∥e_{i}-e_{j}{∥}^{2}+\epsilon ,\;\text{for}\;i,j=1,\ldots ,N,$$

where ϵ is a small constant added for numerical stability.

2) $d_{ij}^{\left(12\right)}$: the squared Euclidean distances between each sequence $e_{i}$ and selected neighbor sequences $e_{j}^{\prime }$, given by:
  $$d_{ij}^{\left(12\right)}=∥e_{i}-e_{j}^{\prime }{∥}^{2}+\epsilon ,\;\;\text{for}\;i,j=1,\ldots ,N.$$

These two distance matrices are then concatenated to form a combined distance matrix $d_{\text{sq}}\in R^{N\times 2N}$:

$$d_{\text{sq}}=[d^{\left(11\right)}|d^{\left(12\right)}].$$

We then convert the distances into Cauchy similarities $\phi_{ij}$, where:

$$\phi_{ij}=\frac{1}{1+d_{{\text{sq}}_{ij}}},\;\text{for}\;i=1,\ldots ,N;\;j=1,\ldots ,2N.$$

Optionally, the distance matrix can be modified using a fitted kernel transformation gist.github.com/NikolayOskolkov, as: $a\cdot d_{\text{sq}}^{b}$ where *a* and *b* are defined by the following curve-fitting process: First, depending on *min_dist* parameter (with a default value of 1.0) a target function f(x,min_dist) assigns a value of 1 to distances below *min_dist* threshold and decays exponentially for larger values. The model function $\frac{1}{1+a\cdot x^{2b}}$ is then fitted to this target function using curve_fit module from scipy library https://github.com/scipy/scipy, optimizing *a* and *b*. Once fitted, the transformation $a\cdot d_{\text{sq}}^{b}$ is applied to the distance matrix in Cauchy similarities $\phi_{ij}$ equation above. This adjustment preserves connections for small distances and controls the decay for larger ones, enhancing how relationships are represented in projection maps.

To correctly apply repulsive forces only between non-neighbors, we construct a mask matrix $M\in {\left\{0,1\right\}}^{N\times 2N}$:

$$M_{ij}=\{\begin{array}{cc} 0, & \text{if}\;\;j=i\;(\text{self}-\text{repulsion}\;\text{within}\;\text{batch})\\ 0, & \text{if}\;\;x_{i}\;\text{and}\;x_{j}\;\text{are}\;\text{neighbors}\;\text{in}\;\text{the}\;\text{batch}\\ 1, & \text{otherwise}. \end{array}$$

Finally, the t-SimCNE loss consists of two main components:

#### 2.4.1 Positive loss

The positive term encourages sequences to be close to their corresponding neighbors:

$$L_{\text{pos}}=-\text{log}\;\sum_{i}\phi_{i,i+N},$$

#### 2.4.2 Negative loss

The negative term ensures that sequences are repelled from all other non-neighboring sequences:

$$L_{\text{neg}}=\text{log}\;\left(\sum_{j=1}^{2N}M_{ij}\phi_{ij}\right),$$

where the mask $M_{ij}$ ensures that repulsion is only applied between non-neighboring sequences.

Thus, the overall loss function is a sum of the positive and negative terms:

$$\text{Loss}=\frac{1}{N}\sum_{i=1}^{N}\left(L_{\text{pos}}+L_{\text{neg}}\right).$$

In addition, users can experiment with the option to normalize the loss by number of neighbors and non-neighbors. This option normalizes positive loss by the number of neighbors and negative loss by the number of non-neighbors for each sequence within a batch. This normalization helps in extreme cases where a sequence has few or no neighbors. It ensures the model does not overly focus on positioning that particular sequence either exceptionally close or far from the rest, maintaining a balanced perspective.

### 2.5 Data loader and training

We used the PyTorch WeightedRandomSampler as our sampling algorithm, which samples from the dataset based on predefined probabilities of each data point. We computed sampling weight as the logarithm of the number of neighbors. This approach prioritizes sequences with higher number of neighbors during sampling and led to improved model performance compared to uniform random sampling.

### 2.6 Training procedure

The training process is structured in three stages as suggested by t-SimCNE authors. In the first stage, both the LM and the linear transformation layer are trained together to better adapt the pre-trained model to input sequences (default 400 epochs). In the second stage, the LM and linear transformation are frozen, and only the projection head is trained (default 100 epochs). Finally, in the third stage, the entire model is unfrozen and trained further (default 400 epochs). This approach offers sensitivity to project-specific sequence patterns, allowing the model to capture subtle differentiating signals. However, depending on size of dataset can be computationally heavy and requires GPU (e.g. NVIDIA A100). As a reference, training on 10 000 sequences typically takes 10–20 min on a single GPU, while 100 000 sequences can require 2–3 h using two GPUs.

### 2.7 Clustering and refinement

To identify clusters on the final map, Leiden algorithm is applied to 2D embeddings. To further refine boundaries and edge cases, for instance, when sequences on the embedding map have an equal probability of belonging to multiple clusters, we prioritize CDR3 similarity to decide on their cluster labels. This is implemented using DenseClus method https://github.com/awslabs/amazon-denseclus, where two separate embeddings of the same dataset are combined by taking their sum to form their union. In our case, the primary 2D embedding is derived from contrastive learning and a secondary embedding represents the features of the CDR3 of sequences. To create the CDR3 features, we first reduce their diversity by categorizing amino acids based on properties like charge and hydrophobicity (generalized CDR3). We then break the generalized CDR3s into k-mer fragments, reduce their dimensionality with PCA, and apply UMAP to achieve a standard 2D representation.

### 2.8 Visualization and interactive exploration

The resulting data is visualized through a web browser based Panel https://github.com/holoviz/holoviz, platform, allowing users to interactively explore the 2D maps generated by deepNGS Navigator. Currently, the platform supports visualizing the final projection map, allowing users to customize the coloring scheme and point size based on various dataset properties, such as CDR annotations, number of neighbors, sequence length, and gene annotations. In addition, users can explore and zoom into different parts of the map, and use lasso or box select tools to choose sequences of interest. These selected sequences can then be visualized using the platform’s integrated MSA viewer or downloaded directly in a table format for further analysis.

### 2.9 Interpreting evolutionary trajectories in 2D maps

Evolutionary trajectories represent the paths that antibody sequences follow as they accumulate mutations, starting from a common germline and diversifying over time (Morgan and Tergaonkar 2022, Abdollahi *et al.* 2023). deepNGS Navigator is powered by Language models (LM) and contrastive learning. LMs originally developed for natural language, they learn context by modeling co-occurrence patterns. By applying to antibody sequences, they learn the “grammar” of b-cell evolution: conserved motifs, tolerated mutations, and co-evolving residues. This results in high-dimensional embeddings that carry rich functional and evolutionary information but they’re hard to interpret directly. Contrastive learning structures this space by pulling neighbor sequences (based on overall edit distance and CDR3 similarity) closer and pushing non neighbors apart. When applied at repertoire scale across millions of sequence pairs, this shapes the embedding space to approximate the topology of antibody diversification. The resulting maps not only separate sequence families into distinct clusters but also capture substructure within clusters: radial expansions from naive or root sequences (early diversification), branching (clonal divergence), and continuous gradients reflecting increasing mutational load across one branch. While this does not replace high-resolution phylogenetics, it offers a scalable and alignment-free way to visualize and approximate evolutionary relationships directly from large repertoire sequence data.

## 3 Results

### 3.1 Clustering antibody sequences from yeast display libraries with FACS labels

**Figure 2.**
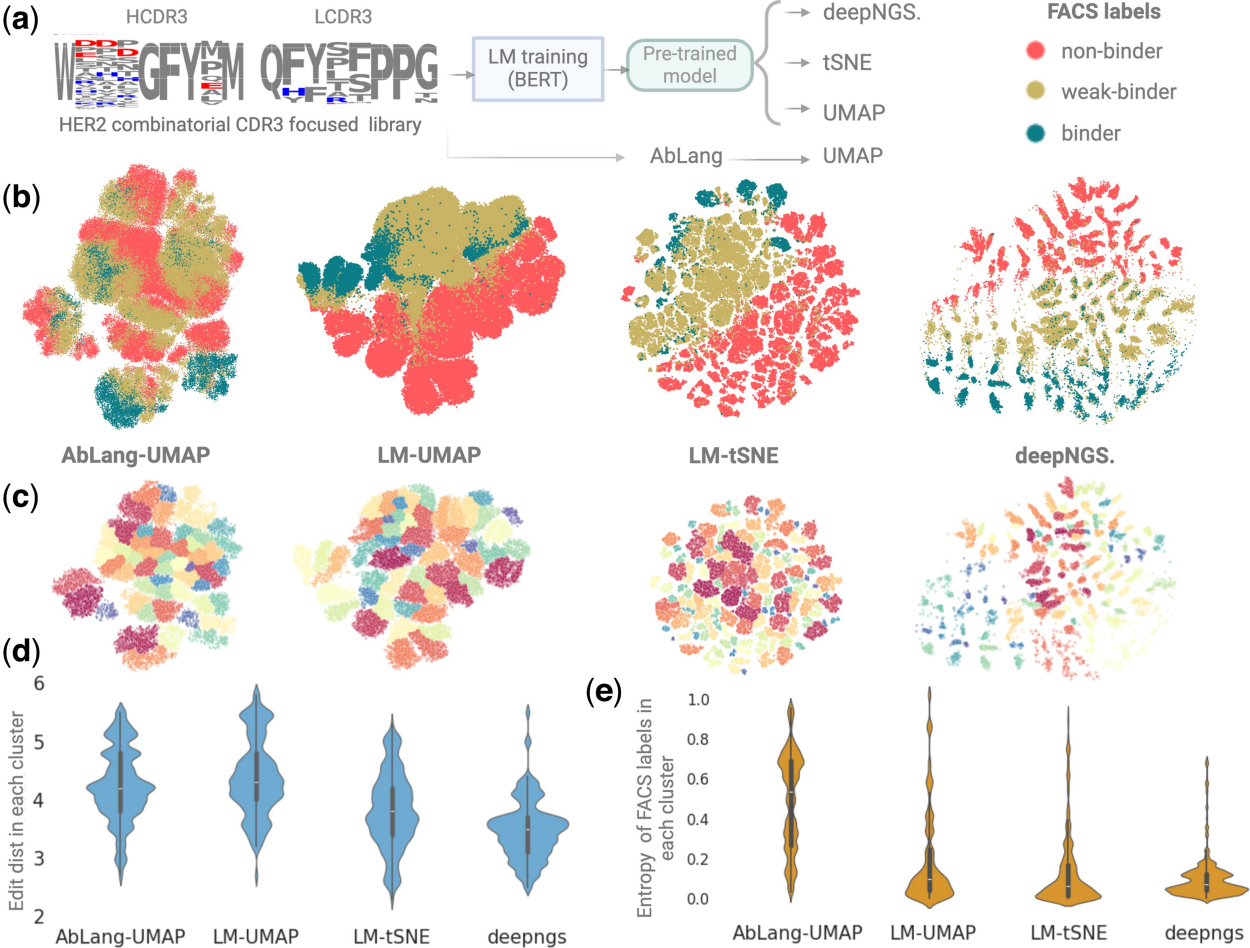
(a) Comparison of clustering methods on the Her2 yeast display dataset. (b) Visualizes the binding FACS labels on 2D projections. (c) Displays the grouped sequences using the Leiden algorithm. The quality of clusters is assessed based on intra-cluster. (d) edit distance and (e) FACS labels entropy. Created with BioRender.com.

Clustering NGS datasets in a way that reflects their relationships and functionality is an important step for efficient hit discovery. If clusters correctly represent the functionality of sequences, we can prioritize diverse selections across map in the initial rounds and shift the focus on optimizing best binders by mining the most promising clusters in subsequent stages. We benchmarked the performance of deepNGS Navigator using 100 000 sampled sequences from Minot *et al.*’s yeast display libraries targeting HER2 (Minot and Reddy 2024). This dataset, labeled by fluorescence-activated cell sorting (FACS) and deep sequencing, provides an ideal dataset for such analysis. In addition to deepNGS Navigator, which trains a custom language model on the input dataset, we explored the impact of contrastive learning on clustering by using the same pre-trained language model with dimensionality reduction techniques like t-SNE (van der Maaten and Hinton) and UMAP. In addition, we evaluated a generalized pre-trained model, AbLang (Olsen *et al.* 2022), combined with UMAP to compare with deepNGS pre-trained LM on input data. As shown in Fig. 2b, while all methods showed some degree of sensitivity to binding properties of sequences, deepNGS Navigator and LM+t-SNE methods more accurately distinguish between binders, weak binders, and non-binders. To quantify these embedding qualities, we used Leiden algorithm (Traag *et al.* 2019) for clustering them, applying 20 neighbors as the hyperparameter across all methods. The resulting number of clusters were: 100 clusters for deepNGS, 125 in LM+t-SNE, and 50 clusters for both LM +UMAP and AbLang+UMAP (Fig. 2c). We evaluated edit distance of sequences and entropy of FACS labels within each cluster, where their overall distributions per method is shown in Fig. 2d and e. Ideally, clusters should exhibit minimized edit distance and entropy while globally separating FACS-derived labels across clusters. deepNGS Navigator consistently demonstrated this trend, validating its high-resolution and fine clustering performance both within and across clusters. This benchmark illustrates how different clustering methods can lead to different interpretation of the same sequences space. deepNGS Navigator generated an intuitive map that separates sequences based on their binding functionality while minimizing edit distances within clusters, such map facilitates achieving the primary goal of identifying diverse binders from large datasets. This approach is adaptable to other datasets, where sequences whether single, paired chains, or specific regions like H3 and L3, can be embedded and clustered, producing maps that reflect their biological relatedness and functionality. This enables users to prioritize diverse sequence selections across promising clusters based on different factors. For example some binder enriched clusters are at the same time highly charged, posing a risk of non-specificity. Such factors can be systematically profiled across entire map, guiding strategic sequence selection and experiments.

### 3.2 Evaluating clustering performance on a synthetic dataset with hierarchical structure

**Figure 3.**
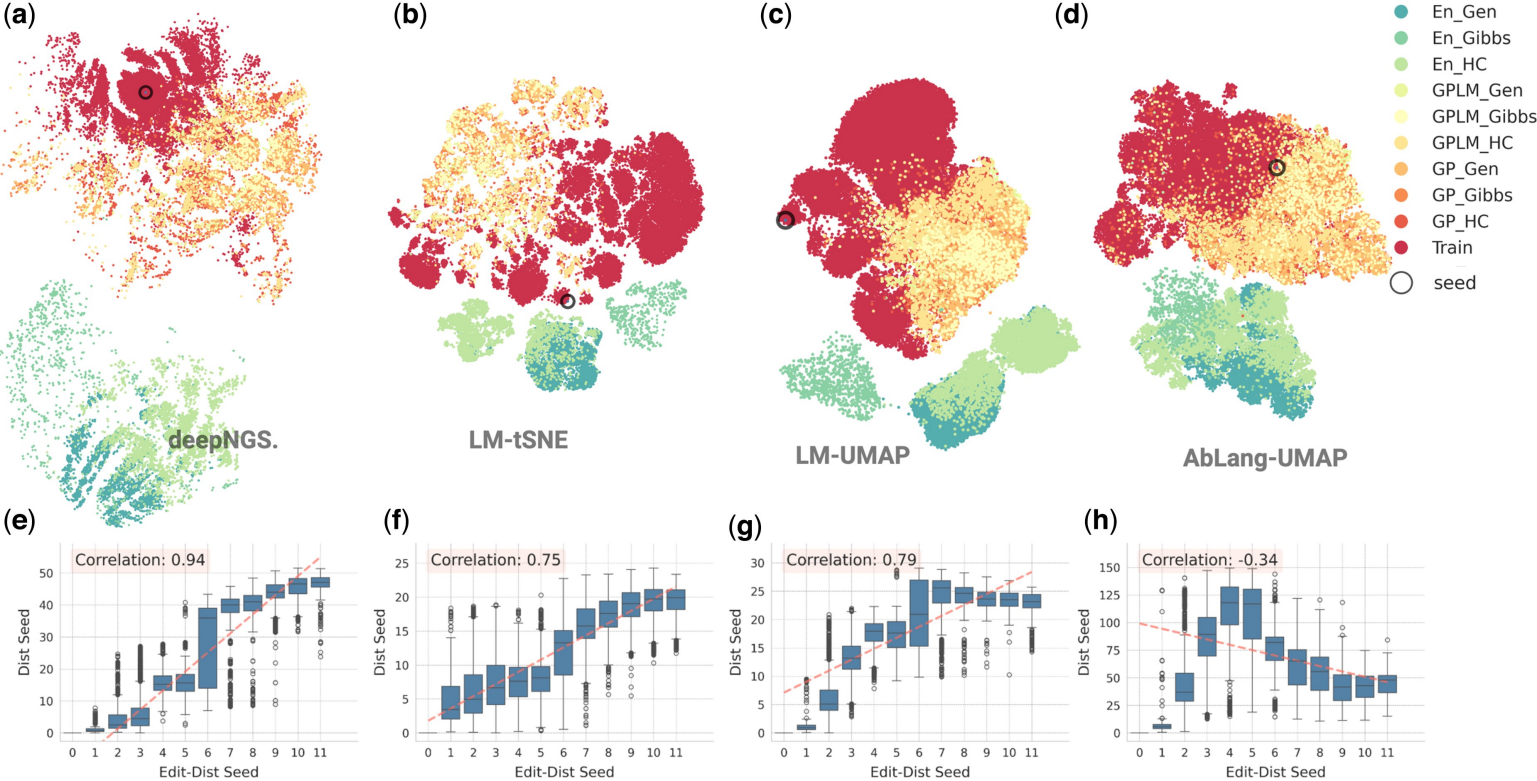
Comparison of different clustering tools on representing a hierarchical synthetic dataset. (a–d) show the 2D embedding colored by origin of sequences, starting with the seed sequence, followed by random low-mutation variants (Train), and designs generated by Gaussian Process (GP) or Ensemble (En) methods, combined with sampling strategies such as hill climbing (HC), genetic algorithm (GA), and Gibbs. (e–h) shows for each method, the correlation between the Euclidean distance from the seed on the map and the edit distance from the seed (ranging from 0 to 11) for each sequence. Created with BioRender.com.

To investigate how well the global structure of a dataset is mirrored in a 2D space, we applied deepNGS Navigator to a synthetically designed heavy chain dataset with a known generation logic. This dataset from Li *et al.* (2023) originates from a single seed sequence and expands to ∼60 000 variants through random mutation and advanced ML generative methods including Gaussian Process and Ensemble models, with hill climb, genetic and Gibbs sampling algorithms, denoted as GP-HC, GP-GA, GP-Gibbs, En-HC, En-GA, and En-Gibbs (Li *et al.* 2023). This dataset’s hierarchical structure is reminiscent of an evolutionary tree, with the seed sequence at the root, the sequences with few small random mutations forming immediate branches, and then the designed sequences representing more distant branches. This structure allows us to assess whether our clustering algorithm can effectively distinguish sequences of varying relatedness, akin to differentiating the root and branches of a tree. Beside deepNGS Navigator, we evaluated the performance of alternative techniques as described in the previous section, including deepNGS Navigator’s pre-trained LM combined with t-SNE or UMAP, and AbLang combined with UMAP. In Fig. 3, we compared final maps both qualitatively by coloring sequences with their origin, and quantitatively by analyzing the correlation between their edit distances and Euclidean distances. As shown in Fig. 3a–d, while all methods provide sequence embedding maps of similar global organization, they differed in resolution and accuracy. First, the customized language model in deepNGS Navigator shows more sensitivity to separation of sequences with different origins compared to general antibody LM in AbLang. Furthermore, with a correlation score of 0.94 between the edit distance from the seed and the Euclidean distance on the map from the seed, deepNGS Navigator 2d embedding offers a finer ordering based on a gradual increase in number of mutations. It organizes the seed sequence at the top, positions the random mutant variants around it, and progressively maps sequences designed using the Gaussian Process (GP) method—which have lower edit distances to the seed—extending outward. The most distant sequences were the ensemble (En) designs, reflecting greater divergence. This spatial arrangement aligns with the expected hierarchical relationships among the sequences. Projecting high dimensional space of antibody sequences into 2D maps, while balancing both global and local structure, is crucial for producing a meaningful low dimensional representation of it, however challenging. While it is important to capture broad biological patterns at a global level, the local neighborhoods and trajectories of sequences should reflect fine details, such as specific sequence motifs, and gradual increase of mutational load. In this case study, we demonstrated that deepNGS Navigator effectively maintains both global and local structures within the dataset by clearly separating sequences based on their origin and showing a strong correlation between the edit distance from the seed sequence and the Euclidean distance on the map. This allows meaningful exploration and exploitation both inside and across different clusters.

### 3.3 Mining antibody phage display pools generated by panning

**Figure 4.**
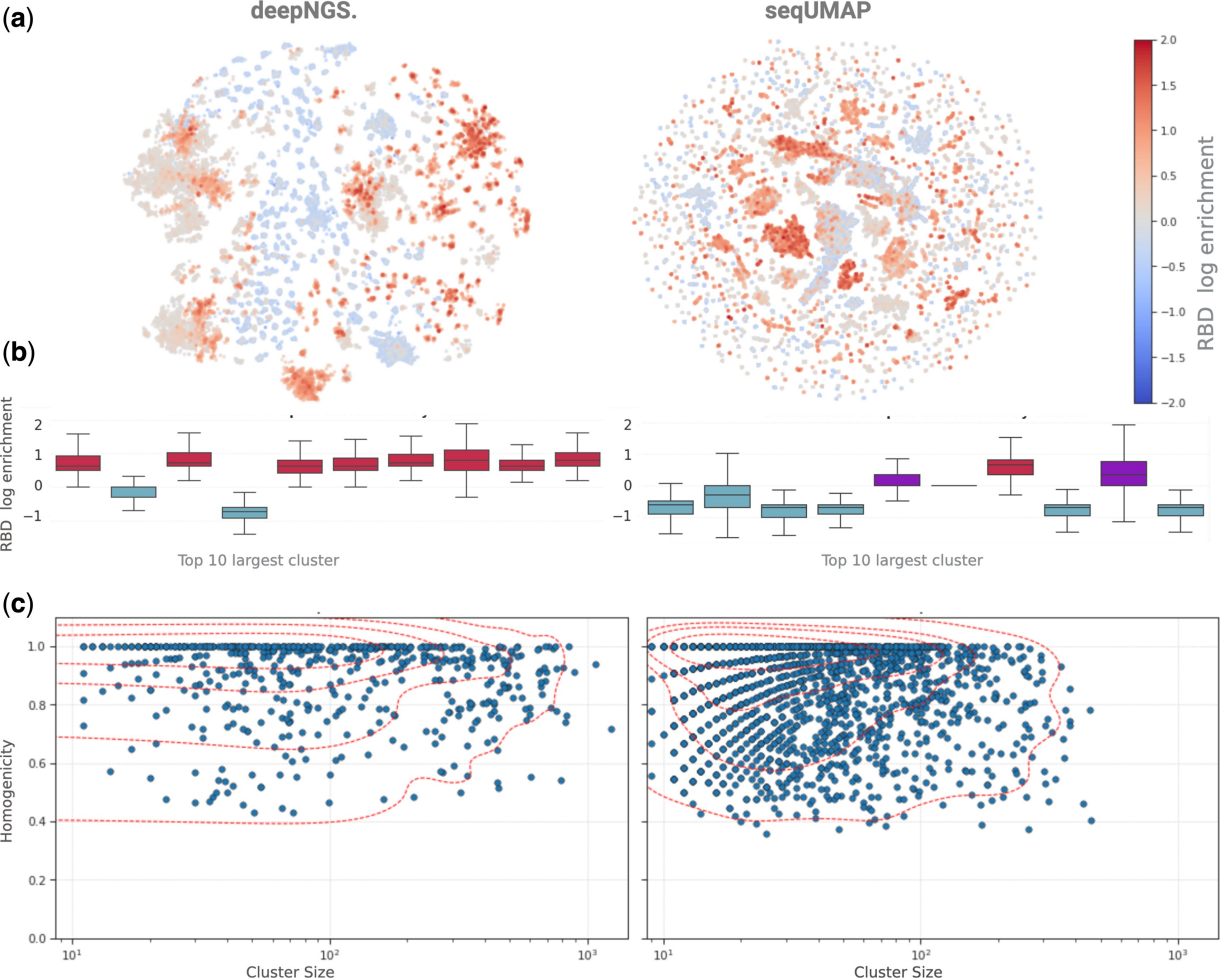
Repertoire analysis using deepNGS Navigator and seqUMAP. (a) 2D embedding maps generated by each method. (b) Enrichment of depletion profile of 10 largest clusters per method. (c) Correlation between cluster size and its homogeneity in terms of depletion or enrichment. Created with BioRender.com.

Display technologies are a crucial element in the hit discovery pipeline for selecting highly specific antibodies. Through multiple panning rounds and conditions sequences that bind to a specific target are enriched. High throughput sequencing (HTS) allows characterizing both final and intermediate panning round sequences. To interpret and leverage NGS with biopanning in depth, efficient computational methods are needed. In this regard, we benchmarked deepNGS Navigator and SeqUMAP using a panning dataset from Hanke *et al.* (2022) This dataset includes ∼96 000 unique antibody sequences obtained from llama immunization targeting the SARS-CoV-2 receptor-binding domain (RBD). Our goal was to identify which tool offers a more accurate and efficient approach for identifying functionally relevant antibody sequences. This is particularly important given the limitations posed by incomplete llama germline databases, which restrict the application of methods like phylogenic tree construction or clonotyping (Hanke *et al.* 2022). As a result, ML based methods that can infer relatedness directly from the data are valuable. SeqUMAP uses a k-mer-based approach to transform sequences into a format suitable for UMAP visualization; here we used seqUMAP with a k-mer size of 3 to analyze dataset. To accommodate variable sequence lengths as input for deepNGS, the sequences were first aligned using AbAlign (Zong *et al.* 2023). Both SeqUMAP and deepNGS Navigator then generated 2D visual maps of the antibody repertoire, with the sequences color-coded according to RBD enrichment levels, where enrichment per sequence is defined as the log ratio of post- to pre-panning frequency, regularized with a pseudocount (Fig. 4a). To assess how effectively each method separated enriched and depleted sequences, we first classified each sequence as enriched (enrichment>0.05), depleted (enrichment<−0.05), or unclear based on their enrichment values, then we applied Leiden clustering algorithm (Traag *et al.* 2019) with 10 neighbors to the 2D embeddings of both methods. SeqUMAP generated ∼1800 clusters, with the largest containing around 300 sequences, while deepNGS Navigator produced 505 clusters, with the largest including more than 1300 sequences. For each cluster, we calculated the homogeneity as the ratio of the dominant enrichment label to the total cluster size. Among the top 10 largest clusters, deepNGS Navigator identified 8 predominantly enriched and 2 depleted clusters. In contrast, seqUMAP’s top 10 clusters included only 1 enriched and 6 depleted clusters, with the remaining clusters displaying mixed enrichment-depletion states. Additionally, a scatter plot showing correlation of cluster size and homogeneity indicates that deepNGS Navigator maintained higher purity despite fewer total number of clusters. In general, larger, fewer clusters are preferred as long as the resulting clusters retain high functional homogeneity. This example highlights deepNGS Navigator’s potential to achieve greater balance in this regard compared to prior methods.

### 3.4 Differentiating naive and memory B cell subpopulations

**Figure 5.**
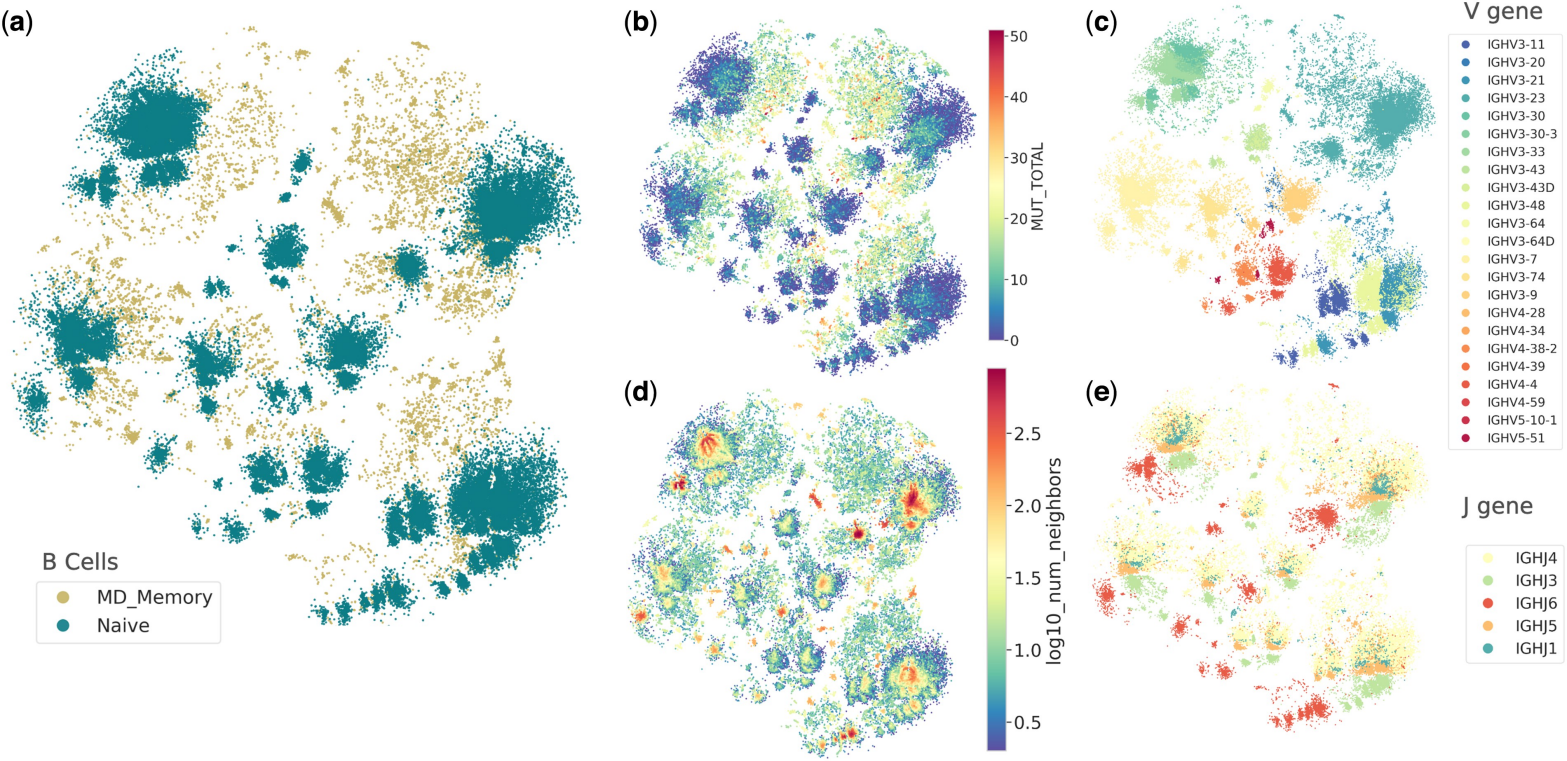
2D projection of deepNGS Navigator map annotated by: (a) B-cell type, (b) number of mutations, (c) V gene, (d) number of neighbors and, (e) J gene distribution. Created with BioRender.com.

Naive B-cells possess a broad range of antigen specificity with moderate affinity. They serve as a reservoir for generating diverse antibody responses upon initial exposures to pathogens and their Fv regions are typically representing germline diversity with the original V(D)J gene sequences without significant mutations. On the other side, Memory B-cells are generated from activated B-cells that have responded to an antigen during a primary immune response, and as a result their Fv regions have undergone somatic hypermutation (SHM), introducing point mutations in the variable regions. To investigate how effectively the characteristics of these two B-cell types are captured by deepNGS Navigator, we collected 150 000 sequences from the study by Ghraichy *et al.* (2024), representing the largest group of sequences with the same length (98 amino acids, starting from HCDR1). As shown in Fig. 5a and b, deepNGS Navigator map captures separation and gradual expansion of naive to memory B-cells, highlighting the gradual increase in mutations underlying evolutionary transition of B-cell types. Furthermore inside clusters, sequences are sorted based on their V gene and J gene (Fig. 5c and e), with those having higher number of neighbors in the center, expanding outward to sequences with fewer neighbors (Fig. 5d). This potentially indicates sequences are ordered based on their evolutionary trajectories.

Using deepNGS Navigator we show the generated dimensionality-reduced projection of the B-cell repertoire preserves the key characteristics of B-cell sequences, allowing to distinguish between naïve and memory B-cell subpopulations. It has been discussed in prior studies that incorporating mutation counts, V and J gene usage, CDR3 physicochemical properties and global repertoire features can similarly distinguish between naïve and memory B-cell subpopulations (Ghraichy *et al.* 2024). Remarkably, in this case, the input was limited to raw sequences, without any additional feature information such as germline annotation. Yet, the resulting map clearly separates sequences by V gene usage (e.g. IGHV3, IGHV4, IGHV5), with further organization by J gene. This highlights the model’s ability to infer biologically meaningful patterns directly from sequence data that reveals transitions of naive to memory B cells, and in addition substructures that provide deeper insight into B cell evolution.

### 3.5 Clonal family inference from large-scale simulated B-cell lineages

**Figure 6.**
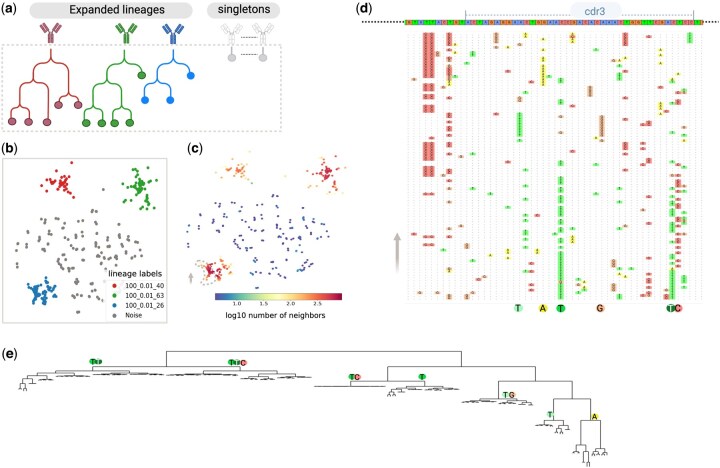
Reconstruction of clonal families from an *in silico*-generated dataset. (a) Schematic representation of the dataset, consisting of 100 lineages with added singleton sequences as noise. Sequences are grouped by total length and CDR3 length before being analyzed using deepNGS Navigator. (b, c) Example output showing sequences of length 367 and CDR3 length of 45, with a 2D embedding map colored by the original family lineage and number of neighbors each sequence has(logarithm base 10). (d) A view of lineage 100-0.01-26, illustrating MSA of the end of FW2 and CDR3 regions for 100 sample sequences along the vertical trajectory marked by a dashed line. The first row displays the consensus sequence, and in subsequent rows deviations from the consensus for each sequence are colored. Key conserved mutations are indicated below the MSA, showing critical mutations that distinguish sub-clusters within the lineage. (e) Construction of a kd-tree using the coordinates of deepNGS map for lineage 100-0.01-26, with CDR3 key mutations mapped onto it. Created with BioRender.com.

The reconstruction of clonal families is a crucial step for understanding adaptive immune response and guiding drug design. During affinity maturation, B-cells originating from a unique V(D)J gene arrangement are expanded and diversified through somatic hypermutation (SHM), each variant competing for stronger binding to an antigen (Chernigovskaya *et al.* 2025). However, accurately identifying these clonal families in highly diverse B-cell repertoires is a significant challenge. Current methods struggle to scale with large datasets and in addition depend on correct V(D)J gene annotation; however many germline databases are incomplete, limiting their application (Hanke *et al.* 2022). In a study by Wang *et al.* (2023) a set of *in silico* datasets were designed to benchmark reconstruction of B cell clonal families using the state of the art methods including: FastBCR (Wang *et al.* 2023), MobiLLe (Abdollahi *et al.* 2022), SCOPe (Nouri and Kleinstein 2018), Partis (Ralph and Matsen 2016), and VJCDR326 (Hershberg and Luning Prak 2015). The simulated datasets includes sequences from different size of lineages generated by various mutation rates, and to make the dataset more realistic, thousands of noise sequences (singletons) were added to each lineage. These datasets resemble clonal expansion and provides a basis for evaluating how well computational methods can identify clonal families (Wang *et al.* 2023). We used largest and most complex set from this study, containing about 40 000 sequences: 20 000 noise sequences and 20 000 forming 100 lineages generated at the highest mutation rate (0.01). This dataset was used to benchmark the performance of deepNGS Navigator in reconstructing clonal families, comparing it to other methods (Fig. 6a).

Unlike previous case studies in this work, where we used protein sequences as input, this dataset consists of nucleotides, allowing us to explore deepNGS navigator’s potential in analyzing DNA sequences. We grouped sequences based on their total length and CDR3 length and applied deepNGS Navigator to each group (31 length groups in total), resulting to 2d maps per length group that further clustered using Leiden method integrated in deepNGS. For example, in Fig. 6, part (b) shows the embedding map for all sequences with a length of 361 and a CDR3 length of 39, color coded by their original family labels, and part (c) shows the same map color coded based on the number of neighbors (logarithm base 10) each sequence has. Using integrated Leiden algorithm we can detect three main clusters with a high number of neighbors, corresponding to the three actual families present in the group. Additionally, some sparse, isolated sequences with few neighbors which match noise sequences. Across all length groups, We compared computed cluster labels with the real labels, and calculated performance measures as described in Wang *et al.*’s paper (Wang *et al.* 2023). To assess the clustering quality of sequences with the same label (excluding noise), we used pairwise metrics across all length groups and identified clusters. A pair was defined as true positive (TP) if both sequences were correctly clustered together in both inferred and original clusters. Similarly, true negatives (TN), false positives (FP), and false negatives (FN) were defined to calculate precision, recall and F1. deepNGS Navigator achieved a high performance, with a precision of 0.99, recall of 0.94, and F1 score of 0.96, matching the best state-of-the-art results (precision: 0.99, recall: 0.93, and F1: 0.97, respectively) without using gene annotation for guidance and instead relying only on sequences.

Furthermore, to specifically assess how well the noise data is handled, clusters categorized into three types: true-inferred clusters (TIC), mix clusters, and noise clusters. True-inferred clusters were those that reconstructed at least 80% of the true family members. Noise clusters consisted solely of noise sequences, while mix clusters were any remaining clusters(Wang *et al.* 2023).

With a TIC fraction of 0.98, a noise cluster fraction of 0.01, and 0.01 mixed clusters, deepNGS Navigator outperforms state-of-the-art methods (TIC: 0.88, mix: 0.12 and noise: 0.00); by producing pure clusters of related sequences while isolating noise sequences. We further focused on a specific lineage (100-0.01-26) as an example, and generated multiple sequence alignment (MSA) along the trajectory of the embedding map (Fig. 6d). In this lineage, diversity was concentrated toward the end of FW2 and in the CDR3 region, as visualized in Fig. 6d. The MSA reveals that as we move along the trajectory, certain patterns remain conserved, indicating that not only different lineages form distinct clusters within the map, but also that meaningful mutational patterns can be identified inside one lineage. Using the embedding coordinates, we constructed a k-d tree (Bentley 1975, Hanke *et al.* 2022) to visualize a phylogeny-like structure of sequences in this lineage (Fig. 6e). By analyzing the consensus at level 3 of this tree, we confirmed that in each sub cluster along the trajectories distinct key residues were consistently preserved. This example demonstrates that embedding maps can be directly utilized to identify closely related sequences or to construct a phylogenetic tree to visualize sequence relationships.

## 4 Discussion

B-cells are key players of adaptive immune system. They can bind and respond to a wide range of antigens by diversifying their receptors through V(D)J gene recombination and further somatic hypermutation (SHM), creating clonal families of B-cells with varying affinity to the antigen (Lindenbaum *et al.* 2021). Identifying these clonal families is a crucial step in drug discovery (Margreitter *et al.* 2018, Lindenbaum *et al.* 2021). Advances in high throughput sequencing (HTS) by generating B-cell receptor (BCR) sequences at an unprecedented scale has provided a much broader perspective on clonal diversity, allowing deeper analysis of immune responses. However, this massive data calls for efficient computational tools capable of unlocking their complexity.

To address this, we developed a method that leverages deep learning-based language models (BERT-style), which have proven highly effective at capturing context and structure in natural languages. Applied to antibody sequences, these models learn the “grammar” of B cell evolution: identifying conserved motifs, tolerated mutations, and co-evolving residues, directly from raw sequences. The resulting high-dimensional embeddings carry rich functional and evolutionary information. To make these embeddings interpretable, we applied contrastive learning, a technique inspired by SimCLR (Chen *et al.* 2020) and t-SimCNE (Böhm *et al.* 2022), and adapted it to the context of antibody repertoires. Contrastive learning structures the 2D embedding by pulling together neighbor sequences (based on edit distance and CDR3 similarity) while pushing apart unrelated ones. When scaled across millions of sequence pairs, this approach reveals a 2D representation of the repertoire that approximates its evolutionary topology. These maps also provide a strong basis for further quantitative analysis. For instance, we assessed global structure preservation by showing strong correlation between edit distance from the seed and 2D Euclidean distance on the map (Fig. 3), or we used distances on the map to construct a tree representing lineages and highlighted the key mutation pattern of each cluster by calculating consensus of clusters (Fig. 6). Therefore, by capturing meaningful relationships between sequences, the 2D embeddings support a wide range of downstream analyses tailored to specific project objectives The pipeline, from training the language models to contrastive learning, generating maps and interactive visualizations with various features, is all integrated into an open-source tool deepNGS Navigator and can be easily accessed by researchers, clinicians, and industry partners.

One of the distinguishing features of deepNGS Navigator is defining neighbor sequences and non-neighbor pairs tailored to the unique characteristics of antibodies, such that users can control allowed diversity in CDR3 regions and full sequence. This customization allows for improved precision around the critical CDR3 regions, capturing subtle but yet important differences in these regions that might be overlooked by algorithms not designed for antibodies.

A better understanding of clonal diversity and sequence relationships in B-cell repertoires can significantly accelerate the hit discovery process by drawing attention to clusters that show stronger antigen responses (Norman *et al.* 2020). These clusters can be detected in various ways depending on the experimental setting. For example, in a bio-panning experiment, clusters with higher enrichment scores will stand out (Mahendra *et al.* 2022). Similarly, in FACS experiments (Fischer *et al.* 2025), clusters that contain mostly binders can be prioritized. While in an immunization campaign (Mahendra *et al.* 2022), embedding naive repertoires alongside sequences from immunized can highlight key differences. Or in case of multiple animal immunizations, embedding sequences from different animals together can help detect any convergence evolution among them. We benchmarked deepNGS Navigator’s performance across several case studies. In one example, using sequences from phage panning of a llama immunized against COVID RBD (Hanke *et al.* 2022), we showed deepNGS Navigator’s clusters retain consistent enrichment or depletion patterns. It outperformed prior methods by producing fewer, larger, and more coherent clusters. In another case study, we applied deepNGS Navigator to embed and cluster sequences of a synthetic library from a yeast display annotated with FACS binding labels to HER2 (Minot and Reddy 2024). The resulting map efficiently separated sequences based on their binding labels while maintaining lower pairwise edit distances and reduced binding label entropy compared to alternative methods. Once promising clusters are identified, a diverse selection representing these clusters will be made to fully leverage the potential of the entire dataset. At the same time, this approach can help to filter out noisy or risky sequences that deviate significantly from the rest of the repertoire, reducing the chance of selecting unreliable candidates.

Understanding the close sequence relatives within each cluster can support the early selection of candidates with fewer developability liabilities during hit discovery. Focusing solely on binding affinity may overlook other critical properties—such as surface charge or hydrophobicity—that can complicate downstream optimization (Li *et al.* 2023). These biophysical features may conflict with affinity-driven mutations, making late-stage adjustments more difficult and resource-intensive. By incorporating these factors earlier in the process, teams can reduce the risk of downstream failures and streamline lead optimization (Teixeira *et al.* 2022). We therefore suggest extending deepNGS Navigator to integrate additional developability metrics, such as immunogenicity risk or aggregation directly into the 2D maps.

deepNGS Navigator’s application is not limited to experimentally derived sequences. With the rise of advanced generative machine learning algorithms (ML), the focus is shifting toward designing *in silico* sequences conditioned on binding to specific antigens and optimizing other biophysical properties. These ML methods can generate vast numbers of sequence designs, raising the question of how to select a diverse and promising subset for testing (Kim *et al.* 2023). deepNGS Navigator can help in this selection process by projecting the sequences onto 2D maps, providing insights into their groups and diversity. We analyzed a synthetic, ML generated sequence set organized in a hierarchical tree structure rooted on a “seed” sequence (Li *et al.* 2023). Our method produced an embedding map that separated sequences by their origin generative method and captured the edit distance relationships more accurately than other approaches. We suggest embedding ML-generated sequences alongside repertoire data to guide selection, with a focus on overlapping clusters and their trajectories to identify natural and promising candidates.

deepNGS Navigator enables analysis of antibody repertoires using only raw sequence data, without relying on V(D)J gene annotations or germline references. This makes it particularly useful for non-model species such as camelids (Hanke *et al.* 2022) and rabbits (Lavinder *et al.* 2014), where annotation is incomplete or affinity maturation follows atypical paths. Unlike clonotyping or tree-based methods that are sensitive to annotation quality (Yermanos *et al.* 2017, 2018), our approach learns directly from sequence patterns using language models, offering a robust alternative. For instance, we demonstrated that deepNGS Navigator captures biologically meaningful structure, separating naive and memory B cells (Ghraichy *et al.* 2024), and clustering sequences by gene usage, without using a germline reference.

The method handles both amino acid and nucleotide sequences. Benchmarking on synthetic nucleotide repertoires showed accurate clonal lineage recovery and superior noise separation compared to existing tools. The learned embeddings also support phylogeny-like analysis, allowing users to query neighbors or reconstruct trees from spatial relationships in the 2D map.

As a promising future direction, we believe that combining sequence-based methods like deepNGS Navigator with structural insights could address some of the limitations inherent in sequence-only approaches. Such hybrid approach could provide a deeper understanding of B-cell evolution and complement ongoing experimental efforts to understand the mechanisms of B-cell affinity maturation (Hsiao *et al.* 2024, Kim *et al.* 2024). While this work focuses on the sequence-based clustering, the integration of structural insights will be the focus of future work on deepNGS Navigator. Finally, we hope that scientists and researchers, will find this tool useful and contribute to its ongoing development. Enhancements such as improving the interactive panel platform by adding features like motif search or filtration could greatly enrich its capabilities.

## Supplementary Material

btaf414_Supplementary_Data

## Data Availability

The deepNGS Navigator source code is publicly available at: github.com/prescient-design/deepngs-navigator and github.com/prescient-design/deepngs-navigator-panel-app.
