# Supplementary material for: deepNGS navigator: exploring antibody NGS datasets using deep contrastive learning
Source: Bioinformatics. 2025 Aug 11;41(9):btaf414. doi: 10.1093/bioinformatics/btaf414 (PMC12448221; doi:10.1093/bioinformatics/btaf414)
Supplement: btaf414_Supplementary_Data [file btaf414_supplementary_data.zip › deepNGS_Navigator_biorxiv_NAR_supplimentary.pdf]

deepNGS Navigator: Exploring antibody NGS datasets using deep contrastive learning

Homa MohammadiPeyhani,[1] Edith Lee,[1] Richard Bonneau,[1] Vladimir Gligorijevic[1] and Jae Hyeon Lee[1,*]

[1]Prescient Design, Genentech
[*]Corresponding author. jaekor91@gmail.com

Abstract

Motivation

High-throughput sequencing uncovers how B-cells adapt in response to antigens by generating B-cell-receptor (BCR) sequences at an unprecedented scale. As BCR datasets grow to millions of sequences, using efficient computational methods becomes crucial. One important aspect of antibody sequence analysis is detecting clonal families or clusters of related sequences, whether they come from immunization, synthetic-libraries or even ML-generated datasets.

Results

We introduce deepNGS Navigator, a computational tool that leverages language models and contrastive learning to transform antibody sequences into intuitive 2D representations. The resulting 2D maps offer a visualization of overall diversity of input datasets, which can be clustered based on the sequence distances and their densities across the map. Beyond grouping related sequences, the 2D maps also represent mutational patterns inferred from sequence embeddings, enabling trajectory analysis and clustering within the projected space. By overlaying properties such as charge, the map helps identify clusters of interest for further investigation while also flagging potentially noisy or non-specific sequences with higher risk. We demonstrate deepNGS Navigator's utilities on several datasets, including: 1) a synthetic-library from a yeast-display targeting HER2, 2) a machine learning-generated dataset with a hierarchical structure, 3) NGS sequences from a llama immunized against COVID RBD, 4) human naive and memory B-cell sequences, and 5) an insilico dataset simulating B-cell clonal lineages.

Availability and Implementation

The deepNGS Navigator source code is available at: github.com/prescient-design/deepngs-navigator and github.com/prescient-design/deepngs-navigator-panel-app.

Contact

jaekor91@gmail.com.

Supplementary Information

Supplementary data, including implementation details and additional figures, are available online.

Key words: Antibody, hit discovery, clustering, deep contrastive learning

Supplementary Material

Supplementary Images

This section provides additional visualizations to support the main text.

deepNGS Panel Application

Once the deepNGS embedding process is completed, each sequence is assigned a set of 2D coordinates stored in the `e1` and `e2` columns. Users can visualize clusters and annotate them using their own tools or libraries of interest. To facilitate exploration, we provide an interactive panel application that allows users to interactively navigate and analyze the 2D embeddings.

Setting Up the Panel Application

To use the panel application, follow these steps as outlined in the README available on GitHub: https://github.com/prescient-design/deepngs-navigator-panel-app

Prepare the Processed File

Ensure that the processed dataset is stored in CSV format. The CSV file should include:

- e1 and e2: Two-dimensional embeddings used for visualization.
- AA: Sequence information that will be used by the panel application to draw MSAs.
- picked_clones: A string identifying clones of interest (e.g., binders). If no such clones are identified, this column can be left null.
- Additional Columns: Other columns in the dataset can be used for point size and color annotations.

Then to integrate input files into the panel application, include their paths and metadata in the processed_files.csv table. This file should contain the following columns:

- **name**: A unique string identifier for the project. Use the format `XXX:YYY` if you want to group several subprojects (e.g., `YYY`) under a main project (e.g., `XXX`) in the panel menu.
- **path**: The file path to the dataset corresponding to the project or subproject.

Run the Panel Application

Start the application using the following command:

```
panel serve main.py --port 5018
```

if the port is not available you can switch to a different number.
Once the application is running, copy the HTTP address provided in the terminal and paste it into your web browser. In the web interface:

1. Select the desired project from the menu.
2. Choose options for point size and color.
3. Press the **Display Map** button.

Exploring the Data

Once the map is displayed:

- Use zoom controls to explore different regions.
- Utilize the lasso or box select tools to highlight sequences of interest.
- View the multiple sequence alignment (MSA) patterns of selected sequences.
- If needed, download selected sequences directly from the interface for further analysis.

This interactive panel application enhances the deepNGS experience by providing an efficient and user-friendly way to analyze and interpret embedding results.

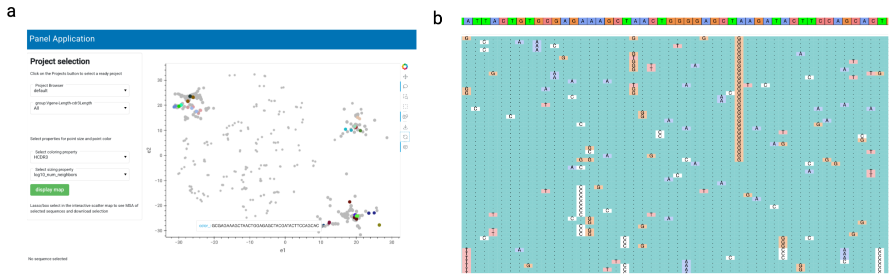

Fig. S1. Demonstration of the panel application on test data. (A) Overview of the interactive interface, where users can select projects and subprojects, define point colors and sizes, and explore the 2D embedding space through zoom and selection tools. (B) Multiple Sequence Alignment (MSA) visualization of the selected sequences, showing variations along the selection trajectory, with colors representing differences relative to the consensus sequence.

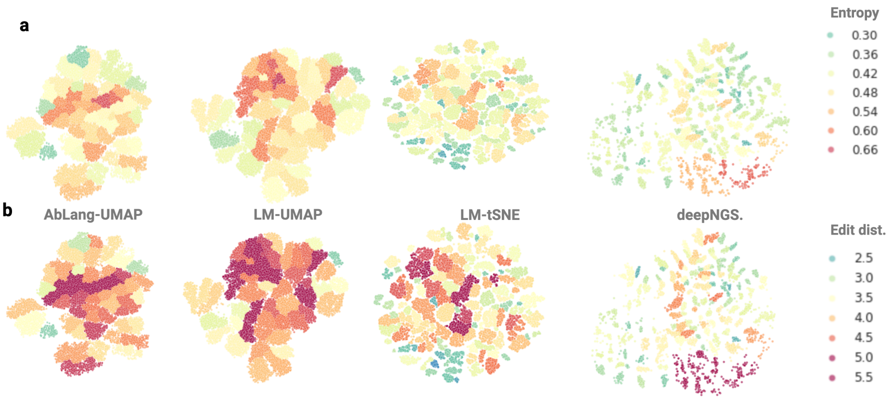

Fig. S2. Visualization of entropy and edit distance distribution across clusters for each method. (a) Displays the per method average entropy within each cluster, with colors ranging from green (lowest entropy) to red (highest entropy). (b) Illustrates the per method average edit distance within each cluster, where green indicates the smallest edit distance and red represents the highest edit distance.
