# Supplementary figures and images for: deepNGS navigator: exploring antibody NGS datasets using deep contrastive learning
Source: Bioinformatics. 2025 Aug 11;41(9):btaf414. doi: 10.1093/bioinformatics/btaf414 (PMC12448221; doi:10.1093/bioinformatics/btaf414)

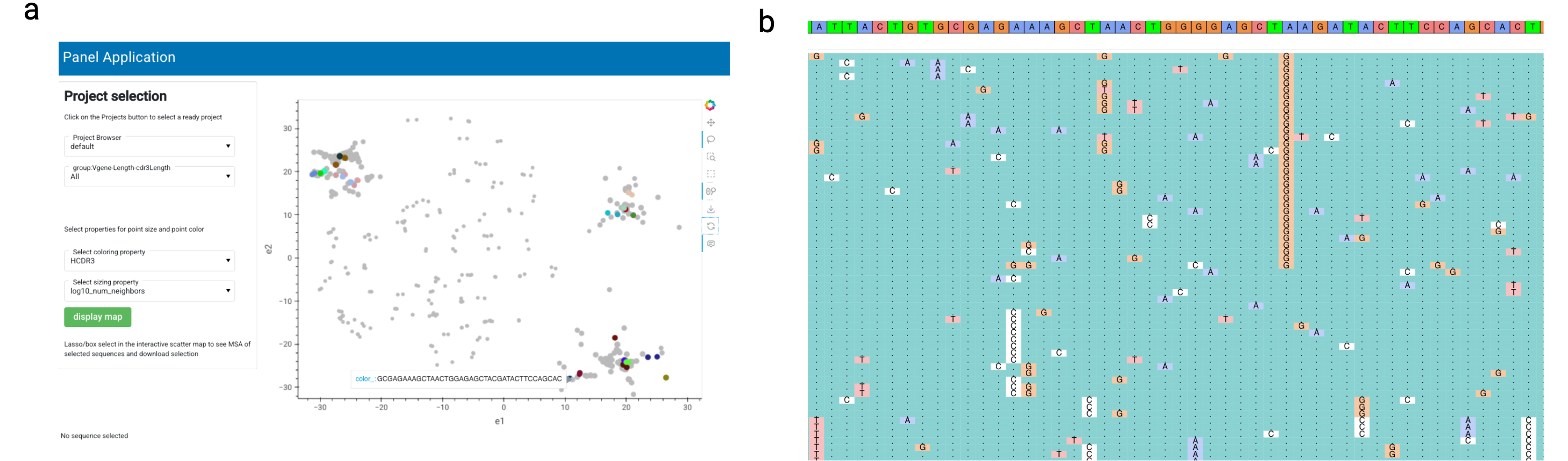

Supplement: btaf414_Supplementary_Data [file btaf414_supplementary_data.zip › FigS1.png]

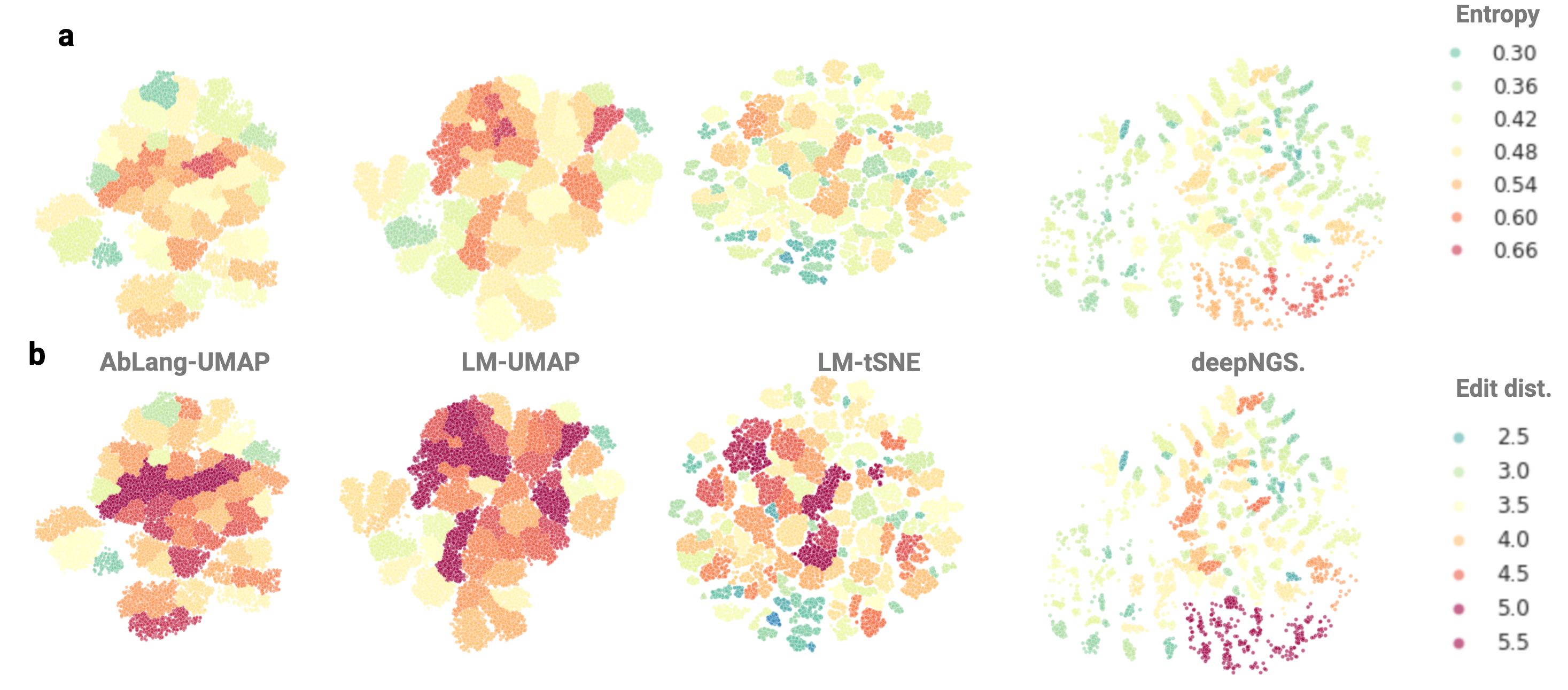

Supplement: btaf414_Supplementary_Data [file btaf414_supplementary_data.zip › FigS2.png]
